# Supplementary material for: Evaluation of Switchgrass Genotypes for Cold-Tolerant Seed Germination from Native Populations in the Northeast USA
Source: Plants (Basel). 2019 Oct 2;8(10):394. doi: 10.3390/plants8100394 (PMC6843356; doi:10.3390/plants8100394)
Supplement: Supplementary file 1 [file plants-08-00394-s001.pdf]

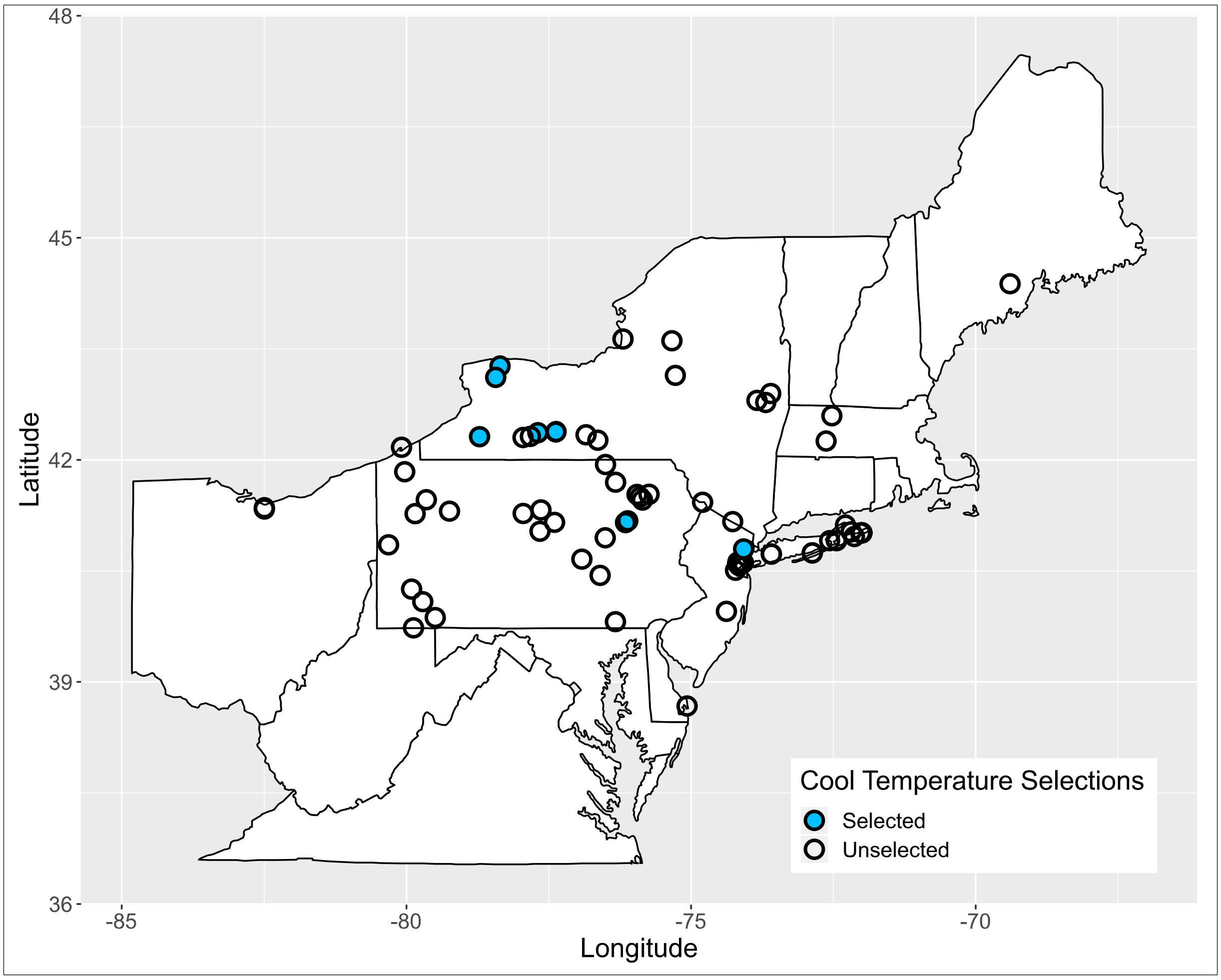

Figure S1: Map of Northeastern USA which shows location of collections for switchgrass accessions comprising 2008 and 2009 breeding nurseries. Circles represent locations where seed was collected to establish the original nurseries in 2008 and 2009 and comprises Cycle 0. Blue circles represent locations from where genotypes were selected for the cold temperature nursery established in 2014.

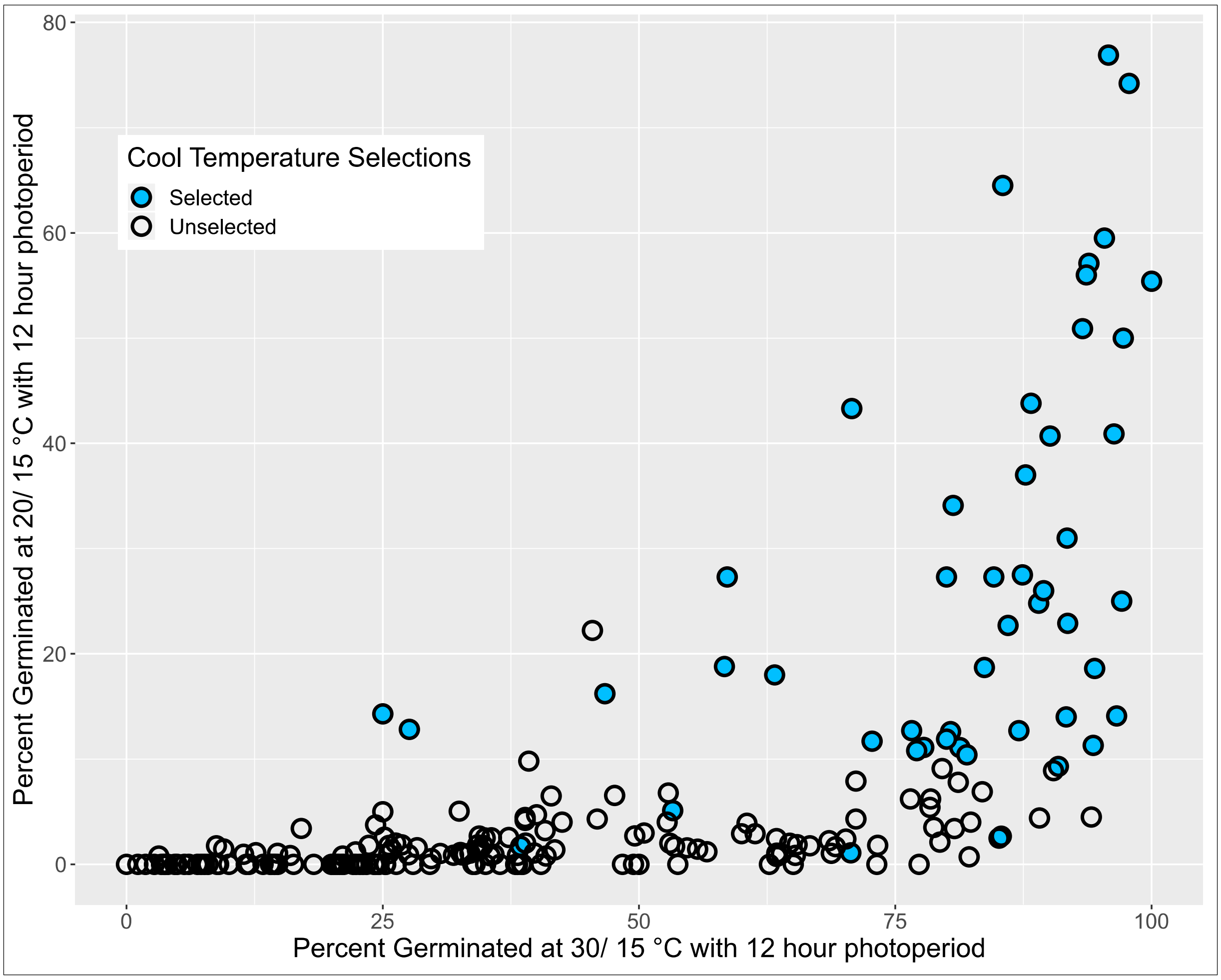

Figure S2: Percent germination of switchgrass genotypes evaluated at two different temperatures from the 2008 and 2009 switchgrass breeding nurseries. Each circle represents seed from one genotype. Blue circles represent genotypes selected for isolation and seed production, while non-colored circles represent unselected genotypes. The progeny of these selected genotypes were planted in the 2014 cold tolerant nursery.

Table S1: Year switchgrass entries were established along with date and location of where seeds were collected in the Northeast, USA. Cycle 0 seed used in experiment 3 was collected from switchgrass genotypes from these nurseries in 2010 and 2011.

Year	Date collected	Location	Year	Date collected	Location	Year	Date collected	Location
2008	10/1996	Luzerne, PA	2008	10/2003	Suffolk, NY	2009	10/2008	Steuben, NY
2008	10/1996	Clinton, PA	2008	10/2003	Suffolk, NY	2009	10/2008	Orleans, NY
2008	10/1996	Susquehanna, PA	2008	10/2003	Oneida, NY	2009	10/2008	Genesee, NY
2008	10/1996	Lackawanna, PA	2008	10/2003	Sullivan, NY	2009	10/2008	Alleghany, NY
2008	10/1996	Crawford, PA	2008	10/2003	Orange, NY	2009	10/2008	Steuben, NY
2008	10/1996	Erie, OH	2008	10/2003	Oswego, NY	2009	NA	Rensselaer, NY
2008	10/1996	Waldo, ME	2008	10/2003	Hudson, NJ	2009	NA	Warren, NY
2008	10/1996	Suffolk, NY	2008	10/2003	Lewis, NY	2009	NA	Saratoga, NY
2008	10/1996	Centre, NY	2008	9/2002	Suffolk, NY	2009	NA	Saratoga, NY
2008	10/1996	Erie, OH	2008	9/2002	Suffolk, NY	2009	NA	Bradford, PA
2008	10/1996	Franklin, PA	2008	9/2002	Suffolk, NY	2009	NA	Bradford, PA
2008	9/1996	Merrimack, NH	2008	9/2002	Suffolk, NY	2009	NA	Schuyler, NY
2008	9/2002	Richmond, NY	2008	9/2002	Suffolk, NY	2009	NA	Wyoming, PA
2008	9/2002	Richmond, NY	2008	9/2002	Suffolk, NY	2009	NA	Wyoming, PA
2008	9/2002	Richmond, NY	2008	10/2002	Suffolk, NY	2009	NA	Luzerne, PA
2008	9/2002	Richmond, NY	2008	10/2002	Suffolk, NY	2009	NA	Clinton, PA
2008	10/2002	Richmond, NY	2008	9/2002	Suffolk, NY	2009	NA	Wyoming, PA
2008	10/2002	Richmond, NY	2008	9/2002	Suffolk, NY	2009	NA	Snyder, PA
2008	10/2003	Richmond, NY	2008	2001	Brooklyn, NY	2009	NA	Columbia, PA
2008	10/2003	Tompkins, NY	2008	10/2002	Franklyn, MA	2009	NA	Clarion, PA
2008	10/2003	Lebanon, NY	2008	10/2000	Essex, MA	2009	NA	Clinton, PA
2008	10/2003	Clarion, NY	2008	10/2000	Hampshire, MA	2009	NA	York, PA
2008	10/2003	Suffolk, NY	2008	10/1991	Sussex, DE	2009	NA	Erie, NY
2008	10/2003	Suffolk, NY	2008	10/1993	MD	2009	NA	Lawrence, PA
2008	10/2003	Suffolk, NY	2009	10/1994	NJ	2009	NA	Venango, PA
2008	10/2003	Suffolk, NY	2009	10/1992	Ocean, NJ	2009	NA	Fayette, PA
2008	10/2003	Suffolk, NY	2009	10/2008	Steuben, NY	2009	NA	Venango, PA
2008	10/2003	Suffolk, NY	2009	10/2008	Steuben, NY	2009	NA	Fayette, PA
2008	10/2003	Suffolk, NY		10/2008	Niagara, NY	2009	NA	Fayette, PA
2008	10/2003	Suffolk, NY		10/2008	Erie, NY			

Table S2: List of accessions planted in 2008 and 2009 switchgrass nurseries selected for germination at cold temperatures. The first column shows the number of genotypes for each accession selected for isolation and seed production. The additional three columns are information about the Parent Germplasm Resources Information Network (GRIN) Plant Name, date collected and origin (County and US State) of each accession, these are the same genotypes indicated by the blue circles in Supplement Figure S2.

Number of Selected Genotypes	GRIN Name	Date collected	Location
1	51966	10/15/1996	Luzerne Co. PA
2	9086098	9/16/2003	Hudson Co. NJ
11	9106189	10/22/2008	Steuben Co. NY
10	9106191	10/8/2008	Niagara Co. NY
1	9106193	10/28/2008	Steuben Co. NY
7	9106194	10/9/2008	Orleans Co. NY
15	9106195	10/8/2008	Genesee Co. NY
